# Comparison of oral and intravenous tranexamic acid in total hip arthroplasty: a systematic review and meta-analysis

**DOI:** 10.1186/s42836-020-00027-7

**Published:** 2020-04-08

**Authors:** Yiming Qi, Yingjuan Li, Chen Wang, Hui Chen, Yunfeng Rui

**Affiliations:** 1 Department of Orthopaedics, Zhongda Hospital, School of Medicine, Southeast University, No. 87 Ding Jia Qiao, Nanjing, Jiangsu 210009 PR China; 2 Multidisciplinary Team (MDT) for Geriatric Hip Fracture Comprehensive Management, Zhongda Hospital, School of Medicine, Southeast University, No. 87 Ding Jia Qiao, Nanjing, Jiangsu 210009 PR China; 3 Orthopaedic Trauma Institute, Southeast University, No. 87 Ding Jia Qiao, Nanjing, Jiangsu 210009 PR China; 4 Trauma Center, Zhongda Hospital, School of Medicine, Southeast University, No. 87 Ding Jia Qiao, Nanjing, Jiangsu 210009 PR China; 5 School of Medicine, Southeast University, No. 87 Ding Jia Qiao, Nanjing, Jiangsu 210009 PR China; 6 Department of Geriatrics, Zhongda Hospital, School of Medicine, Southeast University, No. 87 Ding Jia Qiao, Nanjing, Jiangsu 210009 PR China

**Keywords:** Tranexamic acid, Oral, Intravenous, Total hip arthroplasty, Meta-analysis, Blood loss

## Abstract

**Background:**

Total hip arthroplasty is associated with substantial blood loss which can lead to postoperative anemia. The purpose of this systematic review and meta-analysis was to compare efficacy and safety of oral tranexamic acid (TXA) and intravenous TXA.

**Methods:**

PubMed, EMBASE, and Cochrane Library were searched from inception until December 2019. A combined searching strategy of subject words and random words was adopted. Only clinical randomized controlled trials were included. The comparisons were made with regard to total blood loss, hemoglobin drop, transfusion rate, and postoperative thromboembolic complications including deep vein thrombolism (DVT) and pulmonary embolism (PE). The meta-analysis was conducted by using the Review Manager 5.3, and bias evaluation was performed based on the Cochrane Handbook 5.1.0.

**Results:**

In this meta-analysis, five randomized controlled trials were included. The results showed that there were no significant differences between the oral TXA group and intravenous TXA group concerning total blood loss [mean difference (MD) =3.01, 95% confidence interval (95% CI): − 43.90 to 49.92, *p* = 0.90], hemoglobin drop (MD = 0.05, 95% CI: − 0.10 to 0.20, *p* = 0.50) and transfusion rate of allogeneic blood [risk ratio (RR) =1.09, 95% CI: 0.46 to 2.62, *p* = 0.84]. No significant difference was found in the incidence of thromboembolic events (RR = 1.71, 95% CI: 0.71 to 4.16, *p* = 0.97).

**Conclusions:**

Compared with intravenous TXA, oral TXA is equally able to reduce total blood loss, hemoglobin drop, and transfusion requirement for total hip arthroplasty. It is a lower-cost method that does not increase the incidence of thromboembolic events.

## Background

Total hip arthroplasty (THA) has been demonstrated to be an effective surgical alternative for patients with end-stage hip diseases [1]. More than 500,000 THAs are performed each year in the UK and USA [2].

THA is associated with substantial blood loss which can lead to postoperative anemia [3–6]. Kim et al [4] reported that in the THA procedure, the blood loss ranged between 1000 and 2000 ml, and this increased the requirement of allogeneic blood transfusion. However, allogeneic blood transfusion might increase the risk of adverse effects, such as viral infection, immune response, cardiovascular dysfunction, added cost and even death [7–13]. Recently, the enhanced recovery after surgery protocol has been improved in THA, and the key to the improvement is the blood management [14–16]. A variety of methods have been used to reduce blood loss, including controlled hypotension, regional anaesthesia, autologous blood transfusion, intra-operative blood salvage, and the administration of erythropoietin and antifibrinolytic agents [17, 18].

THA may cause hyperfibrinolysis that may last up to 18 h postoperatively [19]. Lee et al. [18] reported that hyperfibrinolysis may account for 60% of total blood loss. Therefore, attention should be paid to peri-operative application of antifibrinolytics. Antifibrinolytics include Tranexamic acid (TXA), aprotinin and ε-aminocaproic acid, etc.

TXA is a synthetic lysine analog that works by binding to plasminogen and blocking the interaction of plasminogen with fibrin, thereby inhibiting fibrinolysis [20, 21]. This mechanism helps reduce blood loss, but theoretically may also put the patient at an increased risk for thromboembolic events [22]. Recently, multiple studies showed that intravenous TXA during perioperation could effectively reduce blood loss without increasing the risk for thromboembolic events. However, the relevant studies on oral modules are not sufficient, leading to a disagreement over its efficacy and safety.

The purpose of this systemic review and meta-analysis was to verify the efficacy and safety of oral TXA. Our hypothesis was that oral TXA might be as effective and safe as intravenous TXA in patients who undergo THA.

## Materials and methods

### Search strategy

Two reviewers (Yiming Qi and Yingjuan Li) searched PubMed, EMBASE, and Cochrane Library respectively from inception until December 2019. A combined searching strategy of subject words and random words was adopted. The key words, including “THR”, “Arthroplasty, Replacement, Hip”, “THA”, “total hip replacement”, “total hip arthroplasty” and “tranexamic acid” were used in combination. The concrete searching strategy for PubMed was as follows: (((((((THR) OR “Arthroplasty, Replacement, Hip”[Mesh]) OR THA) OR total hip replacement) OR total hip arthroplasty) AND tranexamic acid) AND oral). We included randomized controlled trials (RCTs) that compared oral TXA with intravenous TXA for reducing blood loss or transfusion in patients who underwent THA. Reference lists of all eligible studies and relevant reviews were manually searched for additional studies.

### Eligibility criteria

#### Inclusion criteria were as follows:

1. Patients: adult patients who underwent THA.
2. Intervention: perioperative application of oral or intravenous TXA.
3. Comparison: comparing oral TXA with intravenous TXA.
4. Outcomes: the outcomes concerning efficacy included total blood loss, hemoglobin drop, and transfusion rate; the rate of thromboembolic complications including deep vein thrombosis and pulmonary embolism was chosen as the outcome concerning safety. Total blood loss was selected as the primary outcome.
5. Type of studies: only clinical RCTs were included.

#### Exclusion criteria were as follows

1. Patients: underage patients or adult patients who underwent revision THA or bilateral THA.
2. Intervention: topical administration of TXA.
3. Comparison: no comparison was made between oral TXA and intravenous TXA.
4. Outcomes: total blood loss was not included as an outcome.
5. Type of studies: non-RCTs or concrete description unsuitable for data extraction; basic or laboratory RCTs; letters and comments.

### Data extraction

Two authors (Yiming Qi and Yingjuan Li) independently used the aforementioned search strategy to select the articles from the databases. The titles and abstracts of the articles were reviewed separately. When there was a doubt, the full-text was retrieved for further scrutiny. The included studies were examined thoroughly and independently by two authors (Yiming Qi and Yingjuan Li), and the key data were extracted. Disagreement was resolved by comparing notes or consulting a senior reviewer. The data extracted included author name, country, publish year, sample size, mean age, number of female patients, TXA intervention, prophylactic use of antithrombotics, transfusion criteria and outcome data.

### Quality of included studies

Two reviewers (Yiming Qi and Yingjuan Li) independently assessed the risk of bias of every RCT according to the rules of Cochrane Handbook 5.1.0. We created a ‘risk of bias’ table containing the following 7 items: random sequence generation, allocation concealment, blinding, incomplete outcome data, free of selective reporting and other bias. All the items were described as “low risk of bias,” “unclear risk of bias,” or “high risk of bias.”

### Statistical analysis

Meta-analysis was conducted by using the Review Manager 5.3 software pakage (The Cochrane Collaboration, Oxford, UK). For dichotomous data, the risk ratio (RR) with 95% confidence interval (95% CI) was calculated (the transfusion rate and the occurrence of thromboembolic events), and for continuous variables (total blood loss, hemoglobin drop) the mean differences (MDs) were calculated. Statistical heterogeneity was assessed with the *p* and I^2^ values using the standard Chi-square test. I^2^ > 50% or *p* < 0.1 indicated a significant heterogeneity and a random-effects model was applied for the meta-analysis. Otherwise, a fixed-effects model was used.

## Results

### Search results

**Fig. 1:**
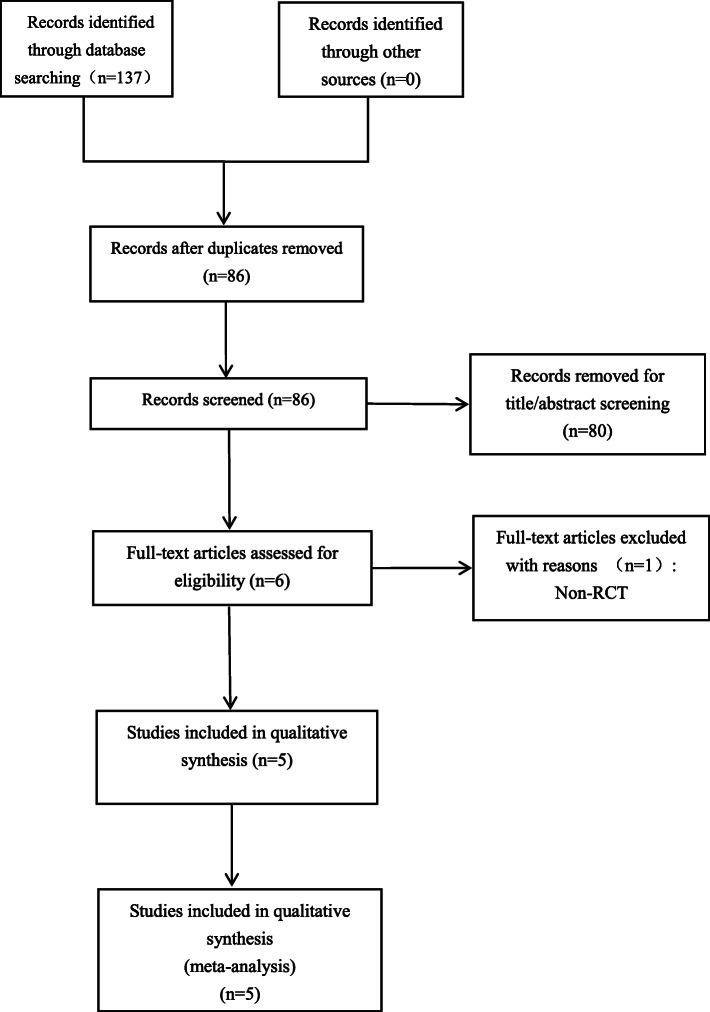
Preferred reporting items for systematic reviews and meta-analyses

A total of 137 studies were identified from the search of the databases and none of them came from other sources. Eighty-six studies remained after the duplicates were removed. Then, we scanned the titles and the abstracts of the 86 citations according to the inclusion and exclusion criteria. As a consequence, 80 citations were excluded. Next, the six remaining studies were carefully full-text-reviewed. Finally five RCTs [23–27] were included in the meta-analysis (Fig. 1).

### Quality of the included studies

**Fig. 2:**
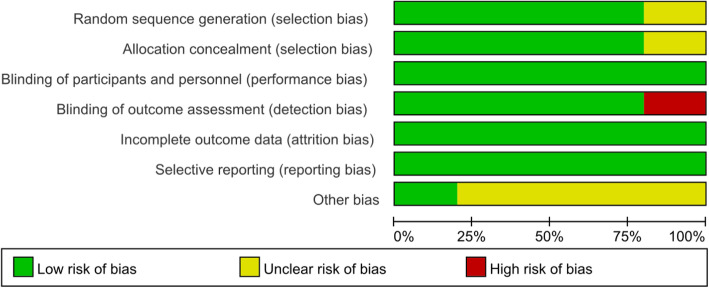
Risk of bias graph

**Fig. 3:**
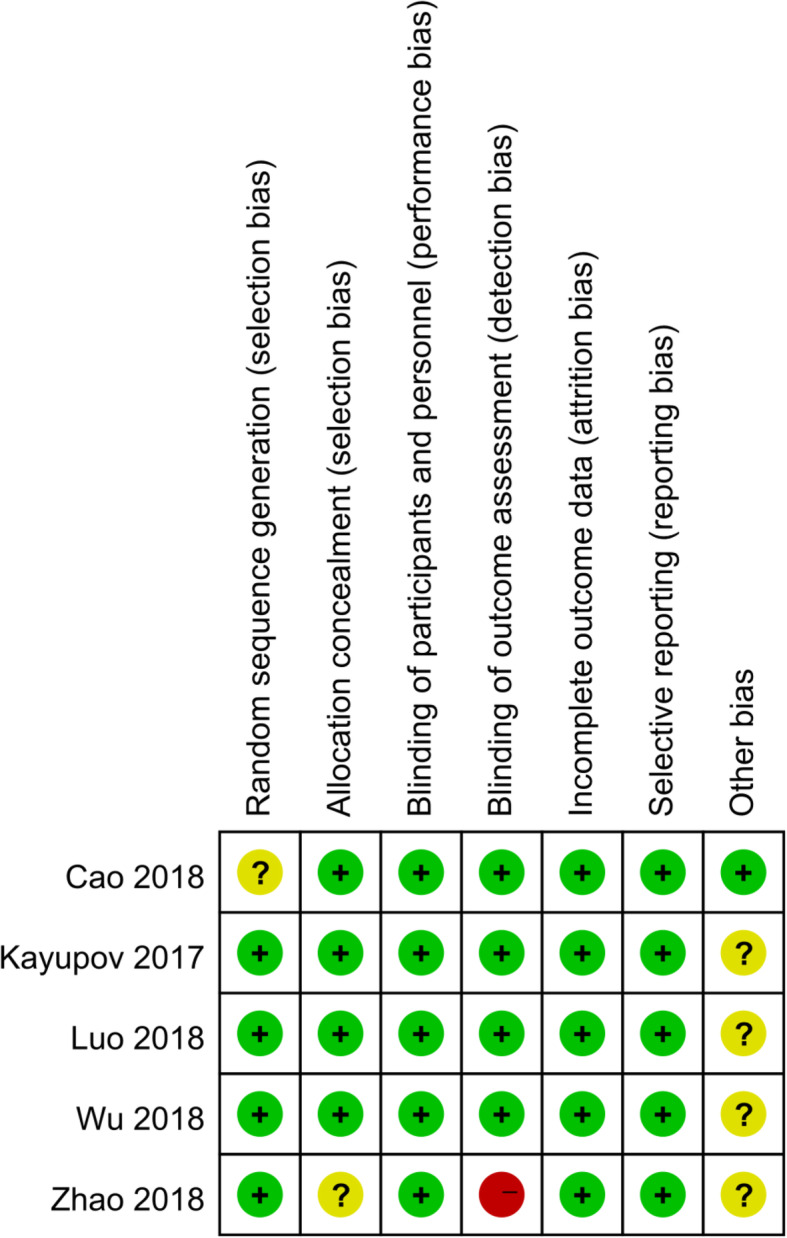
Risk of bias summary

Risk of bias in the included studies is shown in Figs. 2 and 3. For every bias item, the risk is presented as the percentage across all included studies, which indicates the proportion of different levels of risk of bias for each item. Among the included studies, three studies were randomized by computer-generated numbers [25–27], one by random number technique [24], and the remaining one did not report the method of random sequence generation [23]. Four studies conducted the concealment with sealed, opaque envelope [23–26]. Double-blinding was reported in all the five included studies [23–27]. In four studies the outcome assessors were blinded [23–26], and in the remaining one [27], the data analyst was not blinded.

### General characteristics of included studies

All included studies were RCTs published between 2017 and 2018. Four studies were conducted in China and one in the USA. The sample size of the groups ranged from 40 to 60 and the average age of every group varied between 55 to 67.6 years old. As to transfusion indications, in all the studies, transfusion was given when the level of hemoglobin was < 70 g/L or 70–100 g/L with accompanying symptoms of anemia. For deep vein thrombosis prophylaxis, low molecular weight heparin was used in hospital, and in the four Chinese researches, Rivaroxaban was administered after the patients were discharged. Three studies adopted multiple doses [23, 26, 27], and in the other two studies a single dose was applied to every patient [24, 25] (Table 1).

**Table 1:** General characteristics of included studies

Study (Country)	Year	Study type	Sample size O/I	Mean age O/I	Female patients O/I	Oral group intervention	Intravenous group intervention	Prophylactic antithrombotic	Transfusion trigger
Cao (China) [23]	2018	RCT	54/54	55.7/55.7	31/34	2 g of oral TXA 2 h before surgery and 4 h, 10 h, 16 h after surgery	2 g TXA 5-10 min before surgery and 6 h, 12 h, 18 h after surgery	LMWH in hospital Rivaroxaban after discharge	Hb level was < 70 g/L or 70–100 g/L with symptoms of anemia
Kayupov (America) [24]	2017	RCT	40/43	60/55	20/21	1.95 g TXA 2 h before surgery	1 g TXA before surgery	Warfarin	Hb level was < 70 g/L or 70–100 g/L with symptoms of anemia
Luo (China) [25]	2018	RCT	60/60	67.6/67.0	32/33	2 g TXA 2 h before surgery	20 mg/kg TXA 5 min before surgery	Clexane in hospital Rivaroxaban after discharge	Hb level was < 70 g/L or 70–100 g/L with symptoms of anemia
Zhao (China) [27]	2018	RCT	40/40	60.1/59.5	18/17	20 mg/kg 2 h before surgery and 3 h after surgery	15 mg/kg TXA 10 min before surgery and 3 h after surgery	Clexane in hospital Rivaroxaban after discharge	Hb level was < 70 g/L or 70–100 g/L with symptoms of anemia
Wu (China) [26]	2018	RCT	50/50	66.5/65.1	21/20	2 g of oral TXA 2 h before surgery and 3 h, 6 h after surgery	2 g TXA 10 min before surgery and 3 h, 6 h after surgery	Clexane in hospital Rivaroxaban after discharge	Hb level was < 70 g/L or 70–100 g/L with symptoms of anemia

*Abbreviations*: *O* Oral tranexamic acid group, *I* Intravenous tranexamic acid group, *TXA* Tranexamic acid, *Hb* Hemoglobin, *RCT* Randomized clinical trial, *LMWH* Low molecular weight heparin

### Outcomes for meta-analysis

#### Total blood loss

**Fig. 4:**
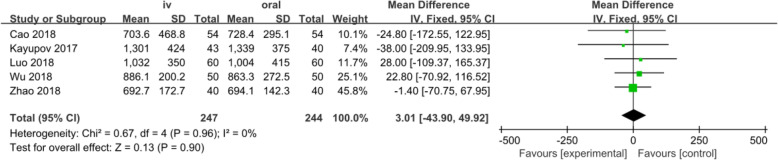
Forest plot for total blood loss

**Fig. 5:**
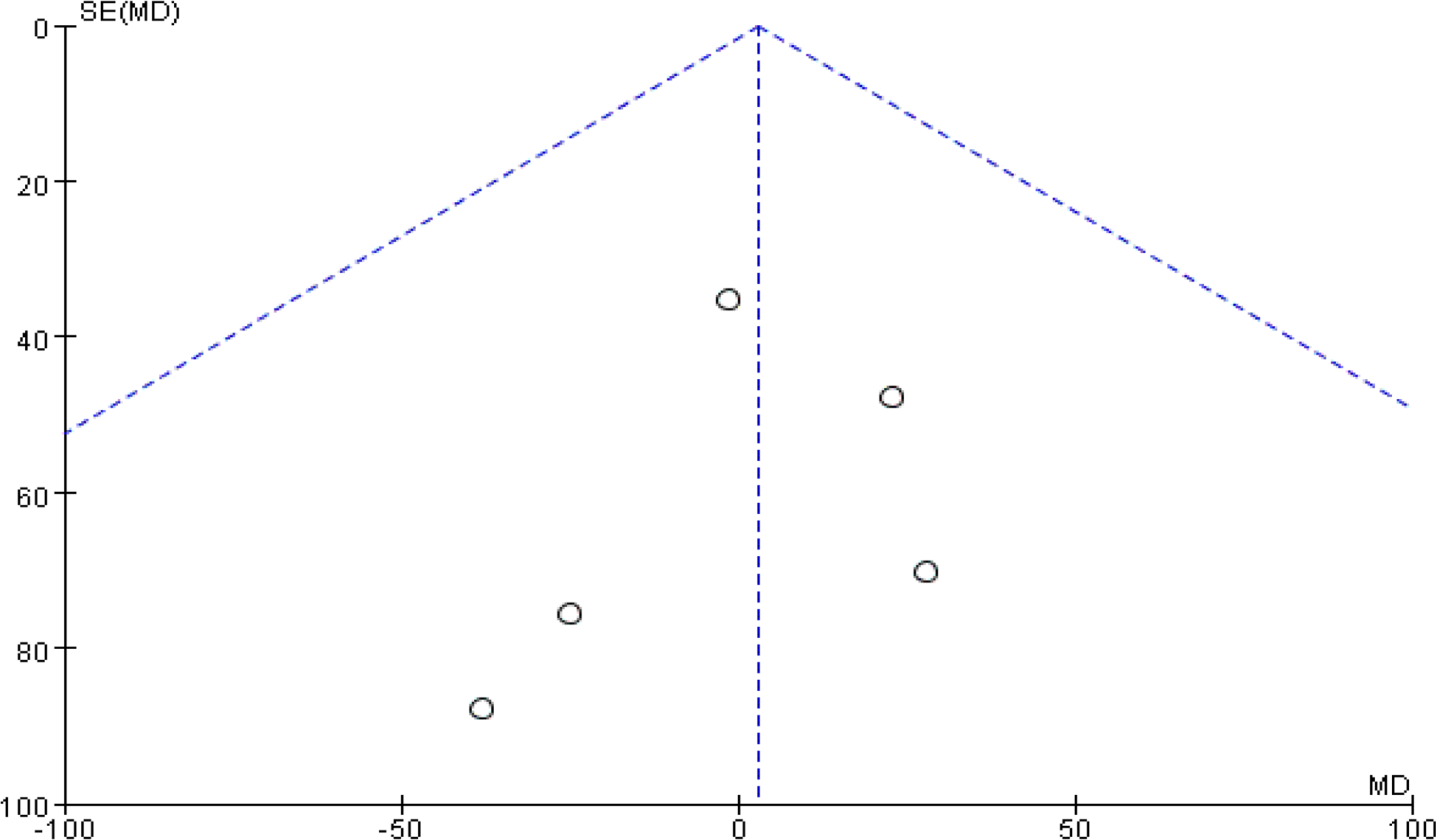
Funnel plot for total blood loss

Five studies involving 491 patients reported the total blood loss. A fixed-effects model was applied because no significant heterogeneity was found among these studies (*p* = 0.96, I^2^=0%). No significant difference was detected in the total blood loss between the two groups (MD = 3.01, 95% CI: − 43.90 to 49.92, *p* = 0.90, Fig. 4). Funnel plot for total blood loss was employed to evaluate publication bias. The funnel plot shows little asymmetry which suggests little publication bias for the meta-analysis of total blood loss (Fig. 5).

#### Hemoglobin drop

**Fig. 6:**
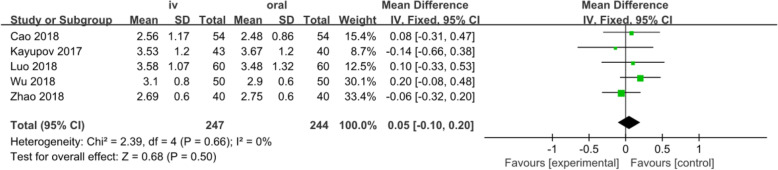
Forest plot for hemoglobin drop

Five studies involving 491 patients reported the post-operation hemoglobin drop. A fixed-effects model was applied because no significant heterogeneity was found among these studies (*p* = 0.66, I^2^=0%). No significant difference was detected in the total blood loss between the two groups (MD = 0.05, 95% CI: − 0.10 to 0.20, *p* = 0.50, Fig. 6).

#### Transfusion rate

**Fig. 7:**
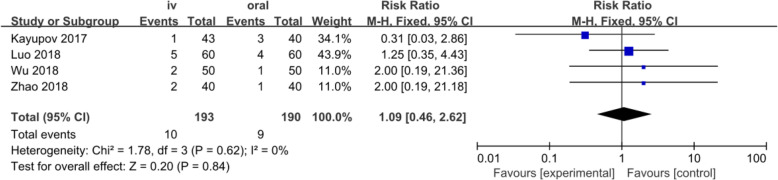
Forest plot for transfusion rate

Four studies involving 383 patients reported the transfusion rate. A fixed-effects model was applied because no significant heterogeneity was found among these studies (*p* = 0.62, I^2^=0%). No significant difference was detected in the transfusion rate between the two groups (RR = 1.09, 95% CI: 0.46 to 2.62, *p* = 0.84, Fig. 7).

#### Thromboembolic events

**Fig. 8:**
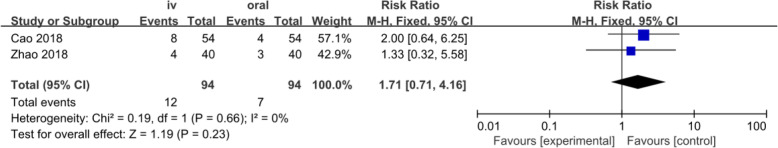
Forest plot for the incidence of thromboembolic events

Two studies involving 188 patients reported the incidence of thromboembolic events. A fixed-effects model was applied because no significant heterogeneity was found among these studies (*p* = 0.66, I^2^=0%). No significant difference was detected in the incidence of thromboembolic events between the two groups (RR = 1.71, 95% CI: 0.71 to 4.16, *p* = 0.23, Fig. 8).

#### Sensitivity analysis and subgroup analysis

No significant heterogeneity was observed in “total blood loss”, “hemoglobin drop”, “transfusion rate”, and “incidence of thromboembolic events”. We excluded one single study to evaluate the influence of the deleted study to the overall result. As a result, omission of any one single study didn’t significantly alter the results, indicating that the results were statistically stable and reliable. No subgroup analysis was conducted for the heterogeneity was low and the number of included studies was small.

## Discussion

Blood loss during and after THA ranged from 700 to 2000 mL, which led to 16 to 37% of the patients requiring transfusions [4, 28, 29]. We found both oral and intravenous TXA yielded similar results in terms of the amount of total blood loss, transfusion rate, and hemoglobin drop. This result is in accordance with a former meta-analysis based on five RCTs about arthroplasties [30]. Evangelista et al [31] reported an elevated risk of deep vein thrombosis and pulmonary embolism after application of TXA. However, several meta-analyses showed TXA might not increase thromboembolic events in patients undergoing THA and the incidence of thromboembolic events was very low [30, 32–35]. Lucas-Polomeni et al [36] reported drug allergy with anaphylactic shock after intravenous TXA. Klak et al [37] reported that topical TXA carried the theoretical risk of periprosthetic infection caused by needle contamination that might even aggravate sepsis. Therefore, the oral form of TXA is considerably safer. Sabbag et al [38] found that, even for patients with a history of VTE, the risk of recurrent VTE (2%) after contemporary THA and TKA was low, and the rate did not increase with the use of intravenous TXA. Besides, we noticed that the patients included in this meta-analysis underwent THA for end-stage hip diseases, such as osteoarthritis, osteonecrosis of the femoral head and developmental dysplasia of hip. Nonetheless, nowadays more and more geriatric patients are suffering from hip fractures, for some of them it may be better to receive THA [39]. Qi et al [40] demonstrated that intravenous TXA reduced total blood loss and transfusion requirements, but did not increase the incidence of thromboembolic events in patients who suffered from hip fractures and underwent THA .

Although Tranexamic acid was discovered more than 50 years ago, the clinical utilization of tranexamic to reduce blood loss and transfusion requirements became popular in the past decade. It was even not included in the 350 essential medicines by the WHO until 2013 [41]. The optimal route, dosage and time for TXA administration in THA remain controversial. In the pooled studies, a loading dose in combination with a following dose or following doses was adopted in three studies [23, 26, 27], and a single bolus dose was administered in two studies [24, 25]. In the two single-dose studies, the average blood loss were more than 1000ml in intravenous and oral TXA groups, and was more substantial than that in the other three studies. Pilbrant et al [42] reported that the bioavailability of oral TXA was only 34% of the same dose of intravenous TXA. In addition, blood TXA was completely eliminated within 8 h. TXA reached a peak 2 to 3 h after oral administration, and the peak plasma level appeared immediately after intravenous administration. The half-life of equipotential doses for the two forms were similar. A TXA plasma concentration of 5 to 10 mg/L has been shown to effectively inhibit fibrinolysis and is considered therapeutic. For oral TXA, it takes approximately 2 h at a dose of 2 g to reach a therapeutic concentration and the level is maintained for approximately 6 h [42]. Some studies showed that hyperfibrinolysis lasted 18 to 24 h postoperatively [43, 44]. Therefore, repeated application of TXA for another 18 to 24 h could further inhibit fibrinolysis, thereby further reducing blood loss. Some studies showed that multiple boluses of oral or IV TXA postoperatively were effective and safe [22, 45–47].

Compared with non-pharmacologic hemostatic agents and even with another pharmacological antifibrinolytic (epsilon-aminocaproic acid), TXA is the most cost-effective medicine to minimize perioperative blood loss in THA [31, 48, 49]. Zhao et al [27] showed that the cost of blood transfusion was much lower with oral TXA (US$ 137 total patients) than with intravenous TXA (US$ 273 total patients) or without application of TXA (US$ 1230 total patients). Cao et al [23] found that, compared with intravenous TXA, oral TXA saved US$ 39 per patient during hospitalization. Wu et al [26] reported that the cost of oral TXA (US$ 88 per patient) was significantly lower than that of intravenous TXA (US$ 463 per patient).

Our met-analysis had several strengths. First, this meta-analysis, comparing oral and intravenous TXA solely in THA, included the latest published RCTs. Second, this meta-analysis was methodologically of high quality since it was conducted in strict accordance with the guidelines of the Cochrane Handbook.

Our study had some limitations. First, only five RCTs were included, and the sample size was small. Second, four out of five studies were conducted in the same setting in China. Third, no subgroup analysis was conducted due to a small number of studies. Larger-sample and high-quality RCTs involving different countries and races are warranted for further verification of the efficacy and safety of oral TXA.

## Conclusions

Compared with intravenous TXA, oral TXA is equally capable of reducing total blood loss, hemoglobin drop, and transfusion requirement for THA. It is a lower-cost alternative that does not increase the risk for thromboembolic events.

## Data Availability

Please contact the authors for relevant data.

## Abbreviations

95% CI: 95% confidence interval
DVT: Deep vein thrombolism
LMWH: Low molecular weight heparin
MD: Mean difference
PE: Pulmonary embolism
RCT: Randomized controlled trial
RR: Risk ratio
THA: Total hip arthroplasty
TXA: Tranexamic acid
